# Chemotherapy‐Induced Myelosuppression in Patients With gBRCA‐m Epithelial Ovarian Cancer: A Retrospective Study

**DOI:** 10.1002/cam4.71724

**Published:** 2026-03-12

**Authors:** Hongtao Hu, Xianglin Nie, Yuxin Jiang, Chaoyi Shi, Xing Chen, Weiyue Zhang, Shan Wu, Yanmei Cheng, Lin Zhang, Yi Jiang, Shulin Zhou, Chengyan Luo, Lin Yuan, Wenjun Cheng

**Affiliations:** ^1^ Department of Gynaecology The First Affiliated Hospital of Nanjing Medical University, Jiangsu Province Hospital Nanjing Jiangsu China; ^2^ Department of Reproductive Medicine Women and Children's Hospital of Ningbo University Zhejiang China; ^3^ Department of Gynaecology The First Affiliated Hospital of Zhengzhou University Zhengzhou Henan China

**Keywords:** chemotherapy‐induced myelosuppression, epithelial ovarian carcinoma, first‐line chemotherapy, gBRCA1/2 mutation

## Abstract

**Background:**

Existing evidence indicates that germline BRCA mutation (gBRCA‐m) may increase chemotherapy sensitivity and toxicity. However, its role in chemotherapy‐induced myelosuppression (CIM) remains unclear. We conducted this study to investigate the influence of gBRCA‐m on CIM incidence and severity in patients with epithelial ovarian carcinoma (EOC).

**Methods:**

Patients with EOC treated at the First Affiliated Hospital of Nanjing Medical University from January 2018 to August 2023 were classified into two groups: gBRCA‐m and gBRCA wild‐type. Chemotherapy regimen and myelosuppression data were retrospectively reviewed. Multivariate analysis assessed the association between gBRCA‐m and CIM incidence and severity in patients with EOC receiving first‐line chemotherapy.

**Results:**

Sixty six (27%) of 242 included patients were gBRCA‐m carriers. The median times to myelosuppression onset and the most severe occurrence were significantly shorter for patients with gBRCA‐m (6.0 vs. 27.0 days, *p* < 0.001; 73.5 vs. 121.0 days, *p* < 0.001). Patients with gBRCA‐m had a greater likelihood of Grade IV (GIV) myelosuppression at onset (aOR = 5.585, 95% CI = 1.621–19.241). During the most severe myelosuppression, patients with gBRCA‐m experienced more pronounced decreases in white blood cells (1.83 × 10^9^ vs. 2.33*10^9^ cells/L, *p* = 0.002), neutrophils (0.73 × 10^9^ vs. 1.08 × 10^9^ cells/L, *p* = 0.001), haemoglobin levels (90.41 vs. 94.14 g/L, *p* = 0.017) and platelets (81.62 × 10^9^ vs. 97.63 × 10^9^ cells/L, *p* = 0.001) and were more prone to febrile GIV myelosuppression (aOR = 2.882, 95% CI = 1.071–7.754). The incidences of chemotherapy dose reduction (aOR = 4.322, 95% CI = 2.048–9.124) and delay (aOR = 6.045, 95% CI = 2.266–16.126) were significantly greater in patients with gBRCA‐m. An analysis across all chemotherapy cycles indicated that patients with gBRCA‐m had greater risks of GIII (aOR = 2.356, 95% CI = 1.770–3.137), GIV (aOR = 2.324, 95% CI = 1.685–3.207) myelosuppression and GIV myelosuppression with fever (aOR = 2.097, 95% CI = 1.077–4.083), as well as a greater incidence of chemotherapy dose reduction (aOR = 2.606, 95% CI = 1.785–3.805) and delay (aOR = 4.118, 95% CI = 2.213–7.663).

**Conclusions:**

EOC patients with gBRCA‐m experienced earlier and more severe CIM, highlighting the need for careful monitoring and tailored management.

**Abbreviations**
BMI: body mass index
BSA: body surface area
CI: confidence interval
CIM: chemotherapy‐induced myelosuppression
EOC: epithelial ovarian carcinoma
gBRCA‐m: germline BRCA mutation
gBRCA‐wt: germline BRCA wild‐type
GIV: Grade IV
HB: haemoglobin
IDS: interval debulking surgery
NACT: neoadjuvant chemotherapy
NE: neutrophil
OR: odds radio
PDS: primary debulking surgery
PLT: platelet
RD: residual lesion
TC: paclitaxel plus carboplatin
TP: paclitaxel plus cisplatin
WBC: white blood cell
XELOX: oxaliplatin plus capecitabine

## 1 Introduction

Ovarian cancer is one of the most lethal gynaecologic malignancies, with epithelial ovarian carcinoma (EOC) being the most common type [1, 2]. The standard treatment involves surgery to remove visible lesions followed by platinum‐based chemotherapy [3]. Initial chemotherapy yields positive responses in approximately 80% of patients with EOC [4, 5]. However, a notable side effect is chemotherapy‐induced myelosuppression (CIM), which often necessitates dose adjustments or treatment delays, potentially inducing chemotherapy resistance and diminishing quality of life [6, 7, 8]. Specifically, febrile neutropenia (FN) increases susceptibility to severe, life‐threatening infections [9, 10]. Therefore, vigilant monitoring of patients' haematologic status post‐chemotherapy is imperative.

Previous studies have indicated that germline BRCA mutation (gBRCA‐m) enhances chemotherapy sensitivity [11, 12], potentially increasing toxicity levels. However, their impact on nontumour cells remains controversial [13, 14]. BRCA proteins are crucial for homologous recombination and DNA break repair, and the defects in these proteins are involved in approximately 15%–28.5% of ovarian cancer cases with gBRCA‐m [4, 15]. In tumours with these mutations, both BRCA alleles are dysfunctional. In contrast, nontumour cells with germline mutations are heterozygous and contain one defective allele [12, 14]. This discrepancy can decrease cellular BRCA protein levels, hampering the repair of chemotherapy‐induced DNA damage and potentially intensifying treatment‐related toxicity.

Research on the relationship between gBRCA‐m status and the risk of developing CIM is limited and has yielded conflicting results [12, 13, 14, 15]. Moreover, related research in the Chinese population is scarce [16]. Understanding gBRCA‐status is crucial for optimising treatment plans and effectively managing side effects. To address this gap, we conducted a retrospective study to investigate the impact of gBRCA mutation status on the incidence and severity of myelosuppression in patients with EOC receiving first‐line chemotherapy.

## 2 Methods

### 2.1 Data Source

The data for this study were obtained from the databases of the First Affiliated Hospital of Nanjing Medical University. Two trained personnel performed the data extraction. This study was performed in accordance with the Declaration of Helsinki and was approved by the Ethics Committee of the First Affiliated Hospital of Nanjing Medical University (reference 2024‐SR‐699). All patients signed informed consent forms.

### 2.2 Study Population

This retrospective study included all patients who underwent ovarian surgery at the First Affiliated Hospital of Nanjing Medical University from January 2018 to August 2023. Patients with pathologically confirmed EOC who underwent BRCA germline gene testing and received first‐line chemotherapy at our hospital were included. Patients who underwent prophylactic salpingo‐oophorectomy or did not receive chemotherapy, as well as those with benign ovarian tumours, borderline ovarian tumours or nonepithelial ovarian malignancies were excluded.

### 2.3 Study Design and Definitions

A retrospective study design was used. Patients were classified into two groups on the basis of their gBRCA mutation status: gBRCA‐m and gBRCA wild‐type (gBRCA‐wt). Potential confounders, such as age at diagnosis, body mass index (BMI), body surface area (BSA), Eastern Cooperative Oncology Group (ECOG) Score, family history of cancer, presence of comorbidities (hypertension, diabetes, hepatitis, immune system diseases or previous cancer history), pathological type, histological grade, tumour laterality, ascites volume, FIGO stage, presence of postoperative residual lesions, cytological examinations of ascites, CA‐125, HE‐4 and relevant haematological indicators before initial treatment, were extracted. BMI was calculated by dividing weight (kg) by height squared (m^2^) and categorised into four groups: < 18.5, 18.5–23.9, 24–27.9 and ≥ 28 kg/m^2^. For FIGO staging, refer to FIGO 2019. BSA was calculated via the Mosteller formula $\mathrm{BSA}\left(m^{2}\right)=\sqrt[{2}]{\frac{\text{weight}\left(\mathrm{kg}\right)*\text{height}\left(\mathrm{cm}\right)}{3600}}$. Ascites status was divided into three groups according to volume: < 100 mL, 100–1000 mL and ≥ 1000 mL. Postoperative residual lesions were categorised as R0 (no visible residual lesions), R1 (residual lesions < 1 cm) or R2 (residual lesions ≥ 1 cm).

The following information was extracted for each chemotherapy cycle: chemotherapy details, including date, cycle, regimen and neoadjuvant status; haematological indicators, including white blood cell (WBC) count (10^9^/L), neutrophil (NE) count (10^9^/L), haemoglobin concentration (g/L) and platelet (PLT) count (10^9^/L) and blood collection date. Other measures included chemotherapy dose adjustments and delays. All patients, including those receiving neoadjuvant chemotherapy, were assigned to receive paclitaxel plus cisplatin (TP), paclitaxel plus carboplatin (TC) or oxaliplatin plus capecitabine (XELOX) once every 3 weeks as first‐line chemotherapy for 6–8 cycles of chemotherapy. Patients at a high risk of recurrence received bevacizumab concomitantly with standard therapy after providing informed consent. All medicines except capecitabine were administered via intravenous infusion. In the chemotherapy phase, patients received paclitaxel (175 mg/m^2^) and cisplatin (75 mg/m^2^) or carboplatin (dose equivalent to an area under the curve [AUC] of 5) and bevacizumab (7.5 mg/kg) on Day 1 of each 3‐week cycle. Patients also received oxaliplatin (130 mg/m^2^) on Day 1 and capecitabine (1250 mg/m^2^/day, bid) on Days 1–14, and this regimen was repeated every 3 weeks. First‐line chemotherapy was continued until cycles had completed, disease progressed, toxicity became intolerable or consent was withdrawn, whichever occurred first. Before each chemotherapy cycle, routine evaluations of complete blood counts, liver and renal function and tumour markers were conducted. Following chemotherapy administration, complete blood counts were monitored every 3 days, and liver and renal function tests were conducted weekly. The test frequency was increased if abnormal results were observed. For example, when myelosuppression occurred, granulocyte‐stimulating therapy was administered, and complete blood counts were monitored daily until the counts returned to above the normal range. Haematologic toxicity was defined according to the National Cancer Institute Common Terminology Criteria for Adverse Events (NCI‐CTCAE) version 5.0 [17].

### 2.4 BRCA1/2 Genetic Test

Blood samples from patients were collected and sent either to AstraZeneca or to the pathology department of our hospital for genetic analysis. Reported variants were confirmed via Sanger DNA sequencing, which was initiated from the original sample using specific genetic primers. Variants were classified in accordance with the regulations of the international ENIGMA consortium (https://enigmaconsortium.org) as previously described in detail [18, 19]. All genetic variants were classified using a 5‐tier variant classification system as proposed by the International Agency for Research on Cancer (IARC) Unclassified Genetic Variants Working Group, namely, deleterious = class 5, likely deleterious = class 4, variant of uncertain significance (VUS) = class 3, likely benign = class 2 and benign = class 1. Class 4/5 germline variants were subsequently defined as ‘variants’ [20]. Patients with VUS were considered noncarriers.

### 2.5 Objectives and End Points

The end points were the time to the occurrence and severity of CIM. The time to the first occurrence of myelosuppression was defined as the period from the start of the first chemotherapy cycle to the first decrease in the WBC, NE and PLT counts and haemoglobin concentration. The time to the most severe myelosuppression was defined as the period from the start of the first chemotherapy cycle to the lowest WBC, NE and PLT counts and haemoglobin concentration during the entire chemotherapy period.

### 2.6 Statistics

Statistical analysis was performed via SPSS v.25.0 (IBM, SPSS Statistics, Armonk, NY, USA). Descriptive statistics were reported as the means and standard deviations or medians and interquartile ranges for continuous variables and as percentages for categorical variables. The chi‐square test or Fischer's exact test was used to compare categorical variables, whereas the Mann–Whitney *U* test or independent *t*‐test was used for continuous variables depending on the distribution of the data. Odds ratios (ORs) with their 95% confidence intervals (CIs) were calculated to evaluate CIM in different gBRCA mutation states via both univariate (unadjusted for covariates) and multivariate statistical methods (adjusted for potential confounders). Variables found to be statistically significant in univariate analysis and confirmed as risk factors in the published literature were entered into the multivariate logistic regression model. *p* < 0.05 was considered to indicate statistical significance.

## 3 Results

**FIGURE 1:**
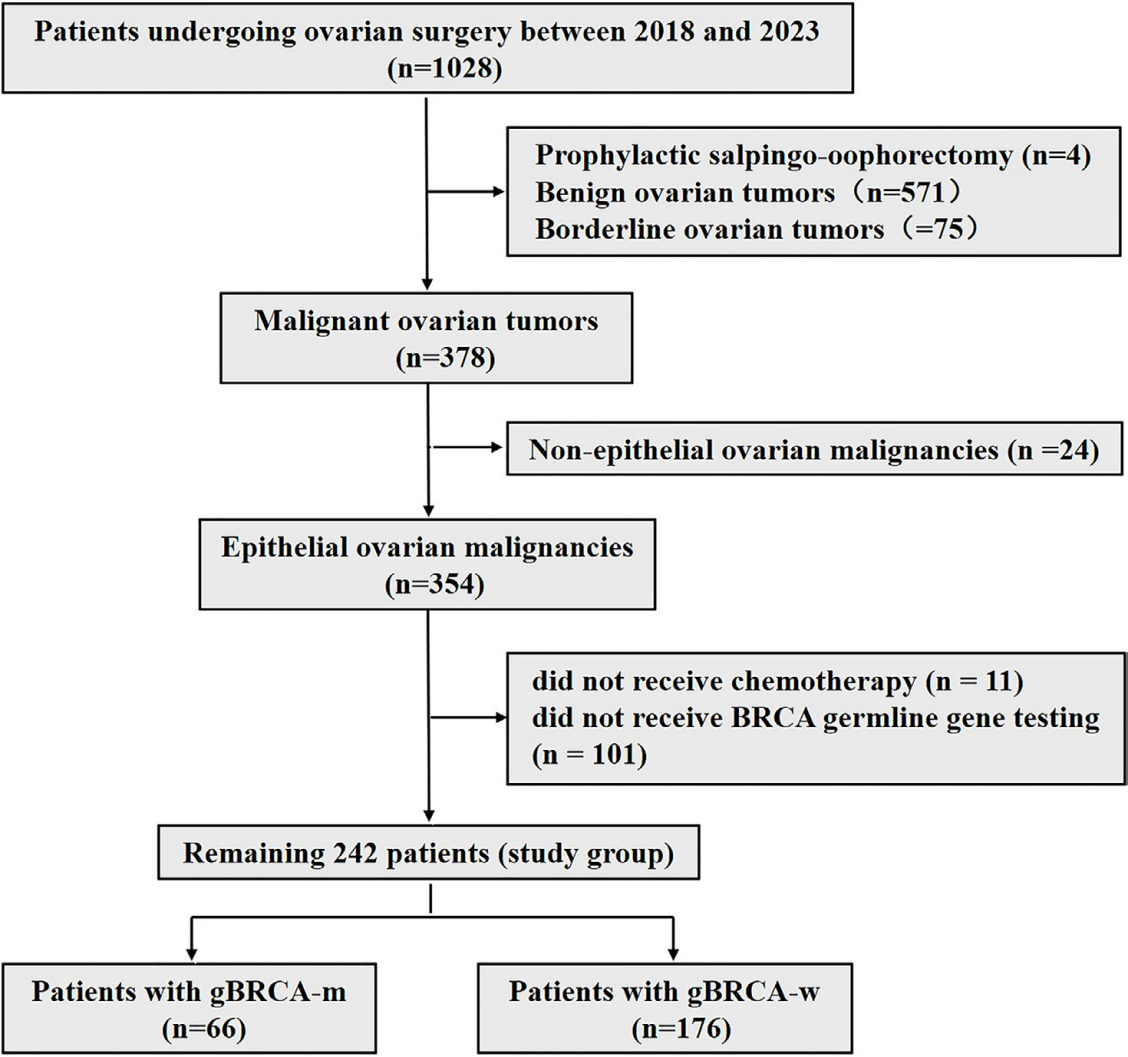
Flow chart showing the study population.

In total, 1028 patients underwent surgery for ovarian conditions at the First Affiliated Hospital of Nanjing Medical University from January 2018 to August 2023, 378 of whom were newly diagnosed with histologically confirmed ovarian carcinoma. After 24 patients with non‐EOC and 11 patients who did not receive chemotherapy were excluded, 343 patients who had histologically confirmed EOC were treated as potential subjects. A total of 242 patients who were subjected to genetic testing were included in the final analysis. Among them, 66 (27%) patients harboured a gBRCA‐m variant and 176 (73%) patients had no such variant (Figure 1).

The baseline characteristics of the patients in the groups are detailed in Table 1. Serous carcinoma was the most common type of tumour, accounting for 88.0% (213/242) of the cases. The gBRCA‐wt group included 11 patients with clear cell carcinoma, 9 with endometrioid carcinoma and 8 with mixed carcinoma, whereas the gBRCA‐m group included only one patient with mixed carcinoma. Twenty one (31.8%) patients in the gBRCA‐m group had a family history of cancer, whereas 35 (19.9%) patients in the gBRCA‐wt group had a family history of cancer. Primary debulking surgery (PDS) was performed in 56.8% (100 patients) of the patients with gBRCA‐wt, a rate that was slightly greater than the 48.5% (32 patients) rate reported in the gBRCA‐m group. The positive cytological rate of ascites was significantly greater in the gBRCA‐m subgroup (69.7%) than in the gBRCA‐wt subgroup (54.5%) (*p* = 0.033). No significant differences were found between the two groups in terms of age, BMI, BSA, ECOG score, hypertension status, diabetes status, hepatitis status, immune system disease status, cancer history, FIGO stage, ascites volume, presence of unilateral tumours, presence of residual lesions, histological grade, number of neoadjuvant chemotherapy cycles, first‐line chemotherapy regimens and bevacizumab treatment (*p* ≥ 0.05).

**TABLE 1:** Baseline characteristics of patients with gBRCA‐wt and those with gBRCA‐m.

Baseline characteristics	gBRCA‐wt (*n* = 176)	gBRCA‐m (*n* = 66)	*p*
Age (years)	55.15 (10.37)	54.42 (9.39)	0.618
BMI (kg/m^2^)			0.698
< 18.5	10 (5.7%)	6 (9.1%)	
18.5–23.9	110 (62.5%)	37 (56.1%)	
24–27.9	48 (27.3%)	19 (28.8%)	
≥ 28	8 (4.5%)	4 (6.1%)	
BSA (m2)	1.58 (0.10)	1.57 (0.12)	0.563
Tumour size (cm)	5.0 (3.0, 8.0)	5.0 (3.5, 8.0)	0.635
ECOG performance status		0.804	
0	24 (13.6%)	8 (12.1%)	
1	132 (75.0%)	53 (80.3%)	
2	17 (9.7%)	4 (6.1%)	
3	3 (1.7%)	1 (1.5%)	
Family cancer history			0.050
Yes	35 (19.9%)	21 (31.8%)	
No	141 (80.1%)	45 (68.2%)	
Hypertension			0.612
Yes	40 (22.7%)	13 (19.7%)	
No	136 (77.3%)	53 (80.3%)	
Diabetes			0.729
Yes	7 (4.0%)	4 (6.1%)	
No	169 (96.0%)	62 (93.9%)	
Immune diseases			0.611
Yes	4 (2.3%)	3 (4.5%)	
No	172 (97.7%)	63 (95.5%)	
Hepatitis			0.616
Yes	15 (8.5%)	7 (10.6%)	
No	161 (91.5%)	59 (89.4%)	
Previous cancer history			0.937
Yes	11 (6.2%)	5 (7.6%)	
No	165 (93.8%)	61 (92.4%)	
NACT			0.246
Yes	76 (43.2%)	34 (51.5%)	
No	100 (56.8%)	32 (48.5%)	
PDS‐IDS			0.189
PDS	100 (56.8%)	32 (48.5%)	
IDS	75 (42.6%)	32 (48.5%)	
No	1 (0.6%)	2 (3.0%)	
Ascites (ml)			0.505
< 100	43 (24.4%)	13 (19.7%)	
100–1000	77 (43.8%)	27 (40.9%)	
> 1000	56 (31.8%)	26 (39.4%)	
RD			0.947
R0	109 (62.3%)	41 (64.1%)	
R1	39 (22.3%)	13 (20.3%)	
R2	27 (15.4%)	10 (15.6%)	
Single‐bilateral lesion			0.318
Single	71 (40.3%)	22 (33.3%)	
Bilateral	105 (59.7%)	44 (66.7%)	
Histopathological type			0.020
Serous carcinoma	148 (84.1%)	65 (98.5%)	
Clear cell carcinoma	11 (6.2%)	0	
Endometrioid carcinoma	9 (5.1%)	0	
Mixed carcinoma	8 (4.5%)	1 (1.5%)	
Histological grade			
Poorly	163 (92.6%)	65 (98.5%)	0.159
Moderately	9 (5.1%)	0	
High	4 (2.3%)	1 (1.5%)	
Cytological examinations of ascites		0.033	
Yes	96 (54.5%)	46 (69.7%)	
No	80 (45.5%)	20 (30.3%)	
FIGO stage			0.140
I	19 (10.8%)	3 (4.5%)	
II	14 (8.0%)	2 (3.0%)	
III	121 (68.8%)	52 (78.8%)	
IV	21 (11.9%)	7 (10.6%)	
Unkonwn	1 (0.6%)	2 (3.0%)	
Thrombosis			0.301
Yes	37 (21.0%)	18 (27.3%)	
No	139 (79.0%)	48 (72.7%)	
Number of NACT cycle	2.88 (1.05)	3.12 (1.34)	0.320
Number of first‐line chemotherapy cycle	6.81 (1.22)	7.05 (1.40)	0.207
Chemotherapy regimen			0.452
TP	8 (4.5%)	5 (7.6%)	
TC	166 (94.3%)	61 (92.4%)	
XELOX	2 (1.2%)	0	
Bevacizumab treatment			0.874
Yes	124 (70.5%)	48 (72.7%)	
No	52 (29.5%)	18 (27.3%)	

Abbreviations: BMI, body mass index; BSA, body surface area; gBRCA‐m, germline BRCA mutation; gBRCA‐wt, germline BRCA wild‐type; IDS, interval debulking surgery; NACT, neoadjuvant chemotherapy; PDS, primary debulking surgery; RD, residual lesion; TC, paclitaxel plus carboplatin; TP, paclitaxel plus cisplatin; XELOX, oxaliplatin plus capecitabine.

Comparisons of the serological indicators before treatment are presented in Table 2. Compared with the gBRCA‐wt group, the gBRCA‐m group presented significantly higher CA‐125 levels (575.2 U/mL vs. 305.6 U/mL, *p* = 0.014) and significantly lower CA‐199 levels (8.03 U/mL vs. 9.84 U/mL, *p* = 0.03). There were no significant differences in the baseline WBC, NE or PLT counts or haemoglobin concentration, HE‐4, CEA, ALT, AST, BUN or Cr levels between the two groups (*p* > 0.05).

**TABLE 2:** Serological indicators before treatment among patients with gBRCA‐wt and those with gBRCA‐m.

Characteristics	gBRCA‐wt (*n* = 176)	gBRCA‐m (*n* = 66)	*p*
WBC	6.27 (1.57)	5.99 (1.61)	0.223
NE	4.16 (1.40)	4.09 (1.51)	0.726
HB	119.73 (10.65)	118.62 (11.97)	0.488
PLT	267.81 (84.93)	266.48 (89.19)	0.915
CA125	305.6 (48.7, 790.7)	575.2 (98.7, 2114.5)	0.014
HE4	147.30 (69.94, 508.00)	136.60 (67.97, 438.05)	0.762
CA199	9.84 (6.07, 18.47)	8.03 (3.51, 14.28)	0.03
CEA	1.29 (0.87, 1.90)	1.13 (0.80, 1.68)	0.199
ALT	14.60 (10.33, 21.88)	14.85 (11.28, 18.55)	0.806
AST	19.70 (16.40, 24.90)	19.35 (15.75, 24.80)	0.612
BUN	4.90 (3.80, 5.60)	4.20 (3.60, 5.40)	0.056
Cr	52.1 (46.6, 62.3)	50.5 (46.3, 56.2)	0.199

Abbreviations: gBRCA‐m, germline BRCA mutation; gBRCA‐wt, germline BRCA wild‐type; HB, haemoglobin; NE, neutrophil; PLT, platelet; WBC, white blood cell.

The influence of gBRCA mutation status on the incidence and severity of CIM in patients with EOC receiving first‐line chemotherapy is displayed in Table 3. Patients with gBRCA‐m status experienced earlier onset and more severe CIM than those with gBRCA‐wt status did. Compared with patients with gBRCA‐wt status, the median time to myelosuppression onset was significantly shorter (6.0 days vs. 27.0 days, *p* < 0.001), and the time to the most severe myelosuppression was also earlier (73.5 days vs. 121.0 days, *p* < 0.001) for patients with gBRCA‐m status. At the first occurrence of myelosuppression, patients with gBRCA‐m were more likely to exhibit a decrease in haemoglobin levels (106.47 g/L vs. 109.81 g/L, aOR = 0.951, 95% CI = 0.919–0.985, *p* = 0.004) and had a greater probability of GIV myelosuppression (aOR = 5.585, 95% CI = 1.621–19.241, *p* = 0.006). In terms of the most severe myelosuppression, the gBRCA‐m group more frequently presented with GIV myelosuppression (aOR = 4.828, 95% CI = 1.119–20.824, *p* = 0.035) and lower levels of vital blood components: WBC counts (1.83 × 10^9^/L vs. 2.33 × 10^9^/L, aOR = 0.550, 95% CI = 0.375–0.809, *p* = 0.002), NE counts (0.73 × 10^9^/L vs. 1.08 × 10^9^/L, aOR = 0.379, 95% CI = 0.204–0.687, *p* = 0.001), haemoglobin levels (90.41 g/L vs. 94.14 g/L, aOR = 0.969, 95% CI = 0.944–0.994, *p* = 0.017) and PLT counts (81.62 × 10^9^/L vs. 97.63 × 10^9^/L, aOR = 0.985, 95% CI = 0.975–0.994, *p* = 0.001). Additionally, patients with gBRCA‐m were more prone to GIV myelosuppression with fever (aOR = 2.882, 95% CI = 1.071–7.754; *p* = 0.036). Consequently, the incidence of drug dose reduction during chemotherapy was significantly greater in the gBRCA‐m subgroup (aOR = 4.322, 95% CI = 2.048–9.124, *p* < 0.001), as was the probability of chemotherapy delay (aOR = 6.045, 95% CI = 2.266–16.126, *p* < 0.001) (Table 3).

**TABLE 3:** Influence of gBRCA mutation status on the incidence and severity of CIM.

Characteristics	gBRCA‐wt (*n* = 176)	gBRCA‐m (*n* = 66)	Univariate logistic regression analysis	*p*	Multivariate logistic regression analysis	*p*
			OR (95% CI)		aOR (95% CI)	
Time to onset of CIM (days)	27.00 (6.00, 58.00)	6.00 (2.00, 22.25)	0.969 (0.955, 0.984)	< 0.001	0.965 (0.949, 0.982)	< 0.001
Number of chemotherapy cycles to first CIM	2.21 (1.45)	1.32 (0.66)	0.386 (0.251, 0.595)	< 0.001	0.307 (0.182, 0.519)	< 0.001
Blood counts at CIM onset						
WBC	3.26 (0.95)	3.07 (1.26)	0.827 (0.614, 1.116)	0.214	0.801 (0.580, 1.106)	0.178
NE	1.84 (0.89)	1.75 (1.11)	0.904 (0.664, 1.230)	0.521	0.825 (0.584, 1.165)	0.275
HB	109.81 (10.75)	106.47 (10.33)	0.971 (0.945, 0.997)	0.032	0.951 (0.919, 0.985)	0.004
PLT	177.38 (67.44)	184.94 (86.20)	1.001 (0.998, 1.005)	0.472	0.999 (0.994, 1.003)	0.588
Initial CIM grade						
I	96 (54.5%)	24 (36.4%)	1		1	
II	55 (31.3%)	23 (34.8%)	1.673 (0.864, 3.240)	0.127	1.334 (0.613, 2.905)	0.468
III	17 (9.7%)	10 (15.2%)	2.353 (0.957, 5.788)	0.062	2.436 (0.839, 7.075)	0.102
IV	8 (4.5%)	9 (13.6%)	4.500 (1.571, 12.888)	0.005	5.585 (1.621, 19.241)	0.006
Time to most severe CIM (days)	121.00 (86.75, 153.25)	73.50 (36.25, 124.25)	0.987 (0.981, 0.993)	< 0.001	0.985 (0.978, 0.991)	< 0.001
Number of chemotherapy cycles at the most severe CIM	5.02 (1.82)	3.71 (1.71)	0.661 (0.554, 0.787)	< 0.001	0.640 (0.525, 0.781)	< 0.001
Blood counts at most severe CIM						
WBC	2.33 (0.99)	1.83 (0.98)	0.586 (0.429, 0.800)	0.001	0.550 (0.375, 0.809)	0.002
NE	1.08 (0.73)	0.73 (0.63)	0.456 (0.288, 0.722)	0.001	0.379 (0.209, 0.687)	0.001
HB	94.14 (13.75)	90.41 (12.46)	0.980 (0.960, 1.001)	0.057	0.969 (0.944, 0.994)	0.017
PLT	97.63 (43.54)	81.62 (36.99)	0.990 (0.982, 0.998)	0.010	0.985 (0.975, 0.994)	0.001
Highest CIM grade				0.021		0.018
I	22 (12.5%)	4 (6.1%)	1		1	
II	59 (33.5%)	15 (22.7%)	1.398 (0.418, 4.674)	0.586	1.657 (0.386, 7.105)	0.497
III	41 (23.3%)	13 (19.7%)	1.744 (0.507, 5.994)	0.377	1.483 (0.323, 6.811)	0.612
IV	54 (30.7%)	34 (51.5%)	3.463 (1.098, 10.921)	0.034	4.828 (1.119, 20.824)	0.035
GIV CIM with fever						
No	161 (91.5%)	55 (83.3%)	1		1	
Yes	15 (8.5%)	11 (16.7%)	2.147 (0.930, 4.953)	0.073	2.882 (1.071, 7.754)	0.036
Drug dose reduction						
No	132 (75.0%)	32 (48.5%)	1		1	
Yes	44 (25.0%)	34 (51.5%)	3.188 (1.765, 5.757)	< 0.001	4.322 (2.048, 9.124)	< 0.001
Chemotherapy delay						
No	164 (93.2%)	50 (75.8%)	1		1	
Yes	12 (6.8%)	16 (24.2%)	4.373 (1.940, 9.857)	< 0.001	6.045 (2.266, 16.126)	< 0.001

*Note:* All aORs adjusted for age, BMI, BSA, family cancer history, histopathological type, cytological examinations of ascites, chemotherapy regimen, CA125, CA199, ALT, AST, BUN and Cr.

Abbreviations: aOR, adjusted odds radio; CI, confidence interval; CIM, chemotherapy‐induced myelosuppression; EOC, epithelial ovarian carcinoma; gBRCA‐m, germline BRCA mutation; gBRCA‐wt, germline BRCA wild‐type; GIV, Grade IV; HB, haemoglobin; NE, neutrophil; OR, odds radio; PLT, platelet; WBC, white blood cell.

As shown in Table 4, an analysis of all chemotherapy cycles revealed that patients with gBRCA‐m had a greater risk of Grade III (GIII) (aOR = 2.356, 95% CI = 1.770–3.137, *p* < 0.001), GIV (aOR = 2.324, 95% CI = 1.685–3.207, *p* < 0.001) myelosuppression and GIV myelosuppression with fever (aOR = 2.097, 95% CI = 1.077–4.083; *p* = 0.026), as well as a greater incidence of drug dose reduction (12.3% vs. 5.1%, aOR = 2.606, 95% CI = 1.785–3.805, *p* < 0.001) and chemotherapy delay (5.6% vs. 1.4%, aOR = 4.118, 95% CI = 2.213–7.663, *p* < 0.001).

**TABLE 4:** Outcome analysis of all chemotherapy cycles among patients with gBRCA‐wt and those with gBRCA‐m.

Characteristics	gBRCA‐wt (*n* = 1199)	gBRCA‐m (*n* = 465)	OR (95% CI)	*p*
Degree of CIM				
≤ II	948 (79.1%)	287 (61.7%)	1	
III	143 (11.9%)	102 (21.9%)	2.356 (1.770, 3.137)	< 0.001
IV	108 (9.0%)	76 (16.4%)	2.324 (1.685, 3.207)	< 0.001
GIV CIM with fever				
No	1177 (98.3%)	449 (96.6%)	1	
Yes	22 (1.7%)	16 (3.4%)	2.097 (1.077, 4.083)	0.026
Drug dose reduction				
No	1138 (94.9%)	408 (87.7%)	1	
Yes	61 (5.1%)	57 (12.3%)	2.606 (1.785, 3.805)	< 0.001
Chemotherapy delay				
No	1182 (98.6%)	439 (94.4%)	1	
Yes	17 (1.4%)	26 (5.6%)	4.118 (2.213, 7.663)	< 0.001

Abbreviations: CI, confidence interval; CIM, chemotherapy‐induced myelosuppression; gBRCA‐m, germline BRCA mutation: gBRCA‐wt, germline BRCA wild‐type; GIV, Grade IV; OR, odds radio.

## 4 Discussion

In this study, we investigated the effect of gBRCA‐status on CIM in patients with EOC. We found that patients with gBRCA‐m experienced earlier and more severe myelosuppression during chemotherapy than patients with gBRCA‐wt did. When GIV myelosuppression occurred, patients with gBRCA‐m were more likely to develop fever, often leading to chemotherapy dose reduction and delays. Our analysis of all the chemotherapy cycles in both groups further confirmed that gBRCA‐m status was associated with an increased risk of developing CIM in patients with EOC. These findings emphasise the need for increased vigilance regarding myelosuppression in patients with gBRCA‐m receiving chemotherapy.

The association between gBRCA‐m status and an increased risk of developing myelosuppression during chemotherapy remains controversial. Some studies have shown no significant link between the two variables. For example, a study involving patients with breast cancer reported no significant difference in the incidence of myelosuppression with fever, delays in chemotherapy or changes in chemotherapy regimens on the basis of gBRCA‐m status [21]. Similar results were reported in a multicentre study on breast cancer [22]. However, a study involving 1171 breast cancer patients reported higher rates of haematologic toxicity (GIII–GIV) in patients with gBRCA‐m treated with taxanes but not with cyclophosphamide or platinum‐containing chemotherapy [12]. Most current research focuses on breast cancer, with few studies on ovarian cancer. In 2018, Joanne et al. reported no significant differences in the NE count, haemoglobin level or PLT count during chemotherapy between 432 ovarian cancer patients with and without gBRCA mutations (*p* ≥ 0.06) [23]. However, in that study, the investigators analysed only the first blood collection for each chemotherapy cycle, whereas in our study, we analysed every blood collection report, providing a more comprehensive assessment. Another study in China indicated that gBRCA‐m status did not increase the incidence of myelosuppression in ovarian cancer patients during initial chemotherapy [16]. Although the study had a large sample size, it lacked comparability between groups, with factors such as patient age, drug dosage and initial renal function potentially influencing the occurrence of myelosuppression [24]. In contrast, in our study, we compared baseline data between groups and excluded confounding factors in the multivariate analysis, making the results more credible.

Our findings revealed that patients with gBRCA‐m experienced earlier myelosuppression during chemotherapy and were more likely to experience a decrease in haemoglobin levels at the first occurrence of myelosuppression. In 2023, Ketty et al. reported that among 447 breast and ovarian cancer patients, those with gBRCA1‐m mutations were more susceptible to myelosuppression within 7–14 days after the initial cycle of chemotherapy [25]. Our results consistently revealed that patients with gBRCA‐m typically experience initial myelosuppression at approximately Day 6. Huszno et al. also reported an increased likelihood of neutropenia following the first chemotherapy cycle in patients with gBRCA‐m [26], although they did not specify a specific time frame. Additionally, Lee et al. [27] reported a higher incidence of anaemia in patients with gBRCA‐m, which aligns with our findings. Our study further revealed that patients with gBRCA‐m have a 3.828‐fold greater risk of GIV myelosuppression than patients without BRCA mutations, along with lower WBC counts, NE counts, haemoglobin levels and PLT counts. Similar conclusions were drawn in a previous study, which indicated that BRCA1 mutation carriers were more likely to experience GIII–IV myelosuppression than gBRCA‐wt carriers were (OR = 3.86) [28]. Furthermore, we found that patients with gBRCA‐m had a 3.322‐fold greater risk of requiring a reduction in chemotherapy dose and a 5.045‐fold greater risk of chemotherapy delays than gBRCA‐wt patients did. A retrospective analysis suggested an increased likelihood of chemotherapy delays in gBRCA‐m individuals (OR = 3.860) [29], while our findings indicate even greater risks, possibly due to racial differences in the study population. Across all chemotherapy cycles, patients with gBRCA‐m were more prone to severe myelosuppression that necessitated dosage reduction and chemotherapy delay.

Our findings suggest that germline BRCA mutations not only influence treatment efficacy but may also increase susceptibility to chemotherapy‐related hematologic toxicity. This underscores the broader principle that molecular alterations can influence both therapeutic benefit and adverse event profiles. In this context, recent advances in ovarian cancer research have identified novel therapeutic targets. For instance, the folate receptor‐α–directed antibody–drug conjugate mirvetuximab soravtansine has shown promising activity in platinum‐resistant ovarian cancer [30, 31], and the MEK inhibitor trametinib has demonstrated improved progression‐free survival in low‐grade serous ovarian cancer [32]. Moreover, PARP inhibitors remain an important maintenance strategy in BRCA‐mutated and homologous recombination–deficient tumours, although their indications are being refined with longer‐term data [33]. Beyond these established therapies, novel agents such as CLDN6‐targeted antibody–drug conjugates [34] and WEE1 inhibitors [35] are under active clinical investigation, further underscoring the expanding landscape of biomarker‐driven strategies.

However, BRCA mutations constitute only one component of the homologous recombination deficiency (HRD) landscape and are insufficient to fully predict treatment response or toxicity risk. Recent studies have identified functional biomarkers that more directly reflect tumour behaviour. For example, RAD51 foci have emerged as a validated predictor of platinum sensitivity and survival in ovarian cancer, with higher foci scores observed in non‐responsive organoid models [36]. Meanwhile, phosphorylated RPA2 (p‐RPA2), a marker of replication stress, has also been shown to identify HR‐proficient tumours that retain platinum sensitivity [37]. Additionally, expression of SLFN11 independently predicts platinum sensitivity and favourable survival in both high‐grade serous and clear cell ovarian carcinomas, regardless of BRCA status [38]. These findings highlight the potential of integrating multidimensional biomarkers—such as RAD51, p‐RPA2 and SLFN11—with BRCA testing to improve prognostic accuracy and guide personalised treatment decisions in ovarian cancer.

**FIGURE 2:**
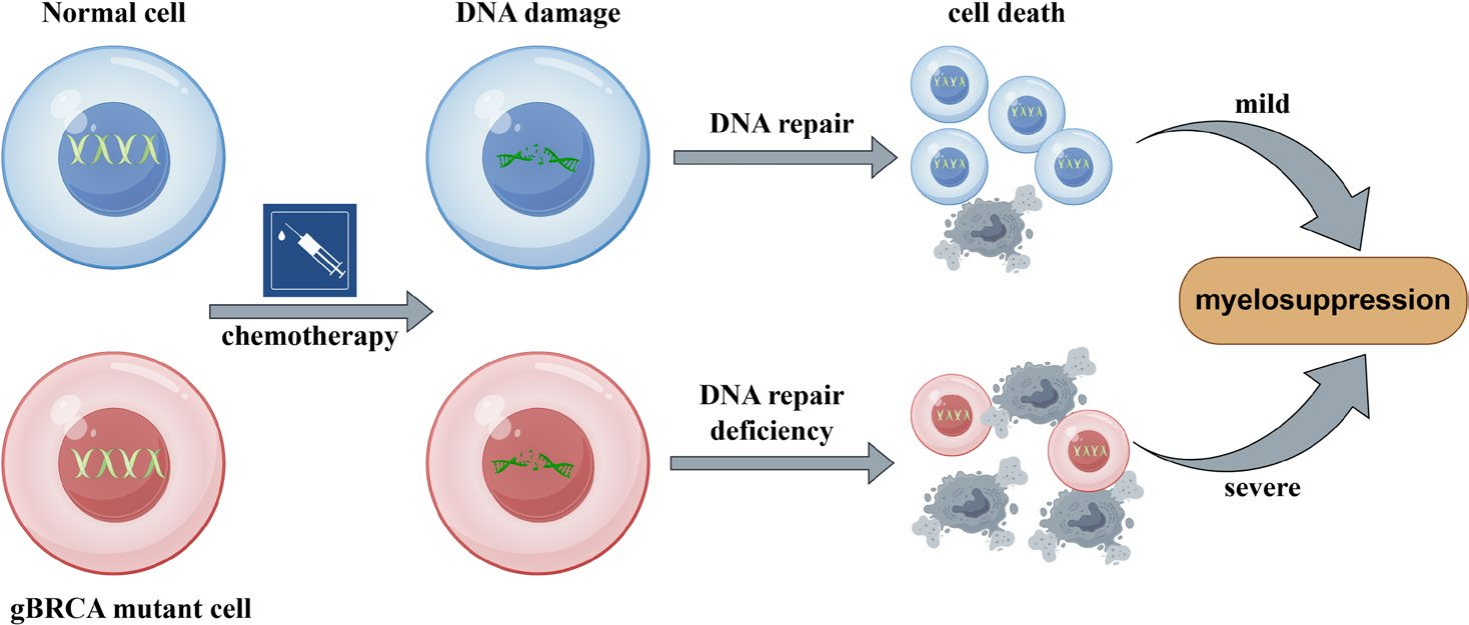
Hypothetical mechanism of germline BRCA mutation and chemotherapy‐induced haematologic toxicity.

In conclusion, these results suggest that patients with gBRCA‐m are more prone to suffer myelosuppression during chemotherapy. This effect may be mediated by impaired DNA damage repair in peripheral blood cells, resulting in increased susceptibility to chemotherapy‐induced myelosuppression. A hypothetical mechanism underlying this association is illustrated in Figure 2. This condition can lead to serious complications, such as anaemia, compromised immune function and increased bleeding tendencies. Moreover, it can significantly impact the treatment process and patient quality of life and potentially prolong hospital stays while increasing medical costs. Therefore, enhancing post‐chemotherapy monitoring for these patients is crucial for promptly detecting and managing potential myelosuppression. The aim of this proactive approach is to minimise complication risks, improve overall treatment efficacy and improve patient quality of life.

### 4.1 Strengths and Limitations

This study represents one of the most comprehensive investigations into the correlation between gBRCA‐m status and the risk of developing CIM in a Chinese population. Our research has several advantages. First, we utilised data sourced from a geographically stable population extracted directly from case notes at the time of treatment, thereby minimising selection and recall biases. Second, by analysing all the chemotherapy cycles, our findings provide a robust evaluation of CIM outcomes. Finally, we rigorously controlled for confounding variables to increase the reliability of our retrospective analysis. However, certain limitations must be acknowledged. The retrospective nature of our data collection method may introduce inherent biases. Additionally, since this study was conducted at a single centre in Jiangsu, the generalisability of our results may be limited to similar local populations within China. Moreover, the relatively small sample size and the heterogeneous distribution of BRCA1/2 variant loci prevented us from performing a locus‐specific analysis of toxicity. Hence, further large‐scale multicentre studies will be essential for performing more specific analyses and providing more robust evidence for the clinical management of ovarian cancer.

## 5 Conclusions

Patients with EOC with gBRCA‐m experience earlier onset and more severe CIM, often leading to chemotherapy dose reduction and delays. These findings highlight the unique challenges faced by patients with gBRCA‐m during chemotherapy and emphasise the need for increased vigilance regarding myelosuppression in patients with gBRCA‐m undergoing chemotherapy. Future research should focus on exploring optimal interventions to improve outcomes in this vulnerable patient population.

## Author Contributions

**Hongtao Hu:** conceptualisation, methodology, investigation, resources, data curation, writing – original draft, visualisation, project administration, funding acquisition. **Xianglin Nie:** methodology, investigation, resources, data curation, writing – original draft. **Yuxin Jiang:** methodology, investigation, resources, funding acquisition. **Chaoyi Shi:** methodology, investigation, resources, funding acquisition, writing – review and editing. **Xing Chen:** methodology, investigation, validation. **Weiyue Zhang:** methodology, data curation. **Shan Wu:** investigation, resources, visualisation, validation. **Yanmei Cheng:** investigation, methodology, resources. **Lin Zhang:** methodology, resources, visualisation. **Yi Jiang:** conceptualisation, investigation, resources, visualisation. **Shulin Zhou:** conceptualisation, investigation, methodology. **Chengyan Luo:** conceptualisation, investigation, methodology, supervision. **Lin Yuan:** conceptualisation, investigation, resources, supervision. **Wenjun Cheng:** conceptualisation, methodology, resources, writing‐review and editing, supervision, project administration.

## Funding

This work was supported by the National Natural Science Foundation of China (82303134 and 82303547), the Youth Talents Development Program of Jiangsu Women and Children Health Hospital (FYRC202004), the Young Scholars Fostering Fund of the First Affiliated Hospital of Nanjing Medical University (PY2021009), Jiangsu Health International Exchange Program sponsorship, and Ningbo Yong Jiang Talent Programme (2021B‐012‐G). The funding agencies played no role in the study design, data collection and analysis, or decision to submit the article for publication.

## Ethics Statement

The authors confirm that the study was performed in accordance with the principles of the Declaration of Helsinki and was approved by the Ethics Committee of the First Affiliated Hospital of Nanjing Medical University (reference 2024‐SR‐699). All patients signed informed consent forms.

## Conflicts of Interest

The authors declare no conflicts of interest.

## Data Availability

The data that support the findings of this study are available on request from the corresponding author. The data are not publicly available due to privacy or ethical restrictions.
